# Structural and functional characterization of dihydrodiol dehydrogenase PahB recognizing high-molecular-weight PAH substrates

**DOI:** 10.1128/aem.00121-26

**Published:** 2026-06-24

**Authors:** Qun Han, Lin-Lin Tian, Lu Guo, Rui Cui, Ze-Shen Liu, De-Feng Li

**Affiliations:** 1 State Key Laboratory of Microbial Diversity and Innovative Utilization, Institute of Microbiology, Chinese Academy of Sciences 85387, Beijing, China; 2 College of Life Sciences, University of Chinese Academy of Sciences 617066, Beijing, China; Universidad de los Andes, Bogotá, Colombia

**Keywords:** dihydrodiol dehydrogenase, high-molecular-weight PAHs, crystal structure, biodegradation

## Abstract

Polycyclic aromatic hydrocarbons (PAHs), especially high-molecular-weight PAHs (HMW-PAHs), are persistent environmental pollutants that are difficult to remove and challenge environmental management. *Altererythrobacter* sp. H2 degrades benzo[a]pyrene, benzo[a]anthracene, pyrene, fluoranthene, and phenanthrene, showing great potential in HMW-PAH removal. The aerobic degradation of HMW-PAHs by this strain is initiated by those well-studied ring-hydroxylating oxygenases, followed by subsequent dehydrogenation by less-studied dehydrogenases. Therefore, we investigated the substrate range and substrate recognition mechanism of dehydrogenase PahB from strain H2. Here, we found that PahB from *Altererythrobacter* sp. H2 can oxidize different HMW-PAH-derived dihydrodiols. Phylogenetic analysis showed that PahB belongs to the NahB-type branch, where it clusters with HMW-PAH-associated homologs, such as BphB-CHY-1, and remains distinct from biphenyl-type BphB dehydrogenases. Crystal structures and docking analyses revealed a hydrophobic, methionine-rich substrate-binding pocket that accommodates different HMW-PAH-derived dihydrodiols in a similar manner. Mutagenesis further showed that methionine residues in this pocket contribute to substrate binding and catalysis. Together, these results define the structural and sequence basis for PahB activity toward HMW-PAH-derived dihydrodiols and expand our understanding of the downstream catabolism of carcinogenic HMW-PAHs.

**IMPORTANCE:**

High-molecular-weight polycyclic aromatic hydrocarbons (HMW-PAHs) are persistent environmental contaminants with well-recognized carcinogenic risks. During aerobic catabolism, cis-dihydrodiol intermediates produced by ring-hydroxylating oxygenases must be oxidized by dihydrodiol dehydrogenases to enter the ring-cleavage pathway, making this second step essential for the overall degradation process. However, no structural information has been available for dehydrogenases capable of processing HMW-PAH intermediates. Previous work has focused mainly on the substrate range of ring-hydroxylating oxygenases, with much less attention to the substrate specificity of downstream dehydrogenases. Here, we elucidate the catalytic and structural basis by which PahB oxidizes dihydrodiols derived from four- and five-ring PAHs, including benzo[a]pyrene. We further show that M219-mediated conformational adaptability is a key structural feature that enables accommodation of bulky substrates beyond the traditional naphthalene- and biphenyl-based systems. These findings expand our understanding of the downstream determinants of HMW-PAH biodegradation and provide a structural basis for engineering dehydrogenases to improve microbial detoxification of carcinogenic PAHs in contaminated environments.

## INTRODUCTION

Polycyclic aromatic hydrocarbons (PAHs), particularly high-molecular-weight PAHs (HMW-PAHs), challenge environmental management and public health due to their extreme hydrophobicity, persistence, toxicity, and carcinogenicity (1). Microbial degradation supports the development of remediation technologies for PAH-contaminated soils and water, and increasing attention has also been paid to enzyme-based approaches for PAH removal (2). The degradation of PAHs is usually initiated by ring-hydroxylating oxygenases (RHOs) under aerobic conditions, which introduce molecular oxygen into the aromatic ring to generate cis-dihydrodiol intermediates (3). Considerable efforts have accordingly focused on identifying and engineering RHOs with expanded substrate scopes (4–6). However, successful environmental degradation depends not only on the initial oxidation step but also on whether the resulting cis-dihydrodiol intermediates can be efficiently converted into downstream ring-cleavage substrates. If this downstream conversion is inefficient or incompatible with bulky PAH intermediates, initial oxidation alone may not lead to productive pathway completion or effective pollutant removal. In contrast, the downstream oxidation of cis-dihydrodiol intermediates by dihydrodiol dehydrogenases has received much less attention, and their substrate recognition and catalytic mechanisms remain poorly understood. These cis-dihydrodiols are then oxidized to the corresponding catechols by dihydrodiol dehydrogenases, which feed into the following ring-cleavage pathways (7).

Until now, all known PAH-related dihydrodiol dehydrogenases belong to NAD^+^-dependent homotetrameric short-chain dehydrogenase/reductase (SDR) family but have diverged into multiple phylogenetic branches (8–11). Biochemical characterization has revealed substantial variation in substrate specificity across different representatives. PahB-OUS82 from *Pseudomonas putida* OUS82 acts on naphthalene dihydrodiol (12). TcbB-P51 from *Pseudomonas* sp. P51 displays an even broader substrate range, including chlorobenzene-, toluene-, biphenyl-, and naphthalene-derived dihydrodiols (13). Similarly, recombinant NahB-G7 from *P. putida* G7 efficiently oxidizes cis-naphthalene dihydrodiol, although its structural data have not yet been published (14). BphB-LB400 from *Paraburkholderia xenovorans* LB400 is involved in biphenyl degradation and acts on cis-biphenyl-2,3-dihydrodiol. Its structure (PDB: 1BDB) reveals the substrate should be accommodated in a hydrophobic cleft near the nicotinamide moiety of cofactor NAD^+^ (15). BphB-B-356 from *Comamonas testosteroni* B-356 oxidizes biphenyl dihydrodiol, and the ternary complex structure of enzyme, NAD^+^, and 4,4′-dihydroxybiphenyl (PDB: 3ZV6) reveals the substrate in a similar hydrophobic pocket to that of BphB-LB400 from *Paraburkholderia xenovorans* LB400 (9, 16). NahB-MC1 from *Pseudomonas* sp. MC1 acts on both naphthalene- and biphenyl-derived dihydrodiols, and its ternary complex structure of enzyme, NAD^+^, and 2,3-dihydroxy-biphenyl (PDB: 5XTG) shows that a flexible loop reshapes the active site to accommodate different PAH dihydrodiols (17). Only BphB-CHY-1 from *Sphingomonas* CHY-1 has been shown to oxidize dihydrodiols originating from high-molecular-weight PAHs, such as benzo[a]pyrene and benzo[a]anthracene (18). Yet, the structural basis for BphB-CHY-1 accommodating bulky multi-ring substrates is unclear, and the reason why different dihydrodiol dehydrogenases have different substrate scopes is not demonstrated.

Here, we performed biochemical and structural characterization of dihydrodiol dehydrogenase PahB from *Altererythrobacter* sp. H2, which is involved in the degradation of high-molecular-weight PAHs. We found that PahB can oxidize different HMW-PAH-derived dihydrodiols, belongs to the NahB-type branch, clusters with HMW-PAH-associated homologs, and does not cluster with biphenyl-type BphB dehydrogenases. It accommodates different PAH dihydrodiols using a methionine-rich hydrophobic substrate pocket, and those methionine residues are essential for HMW-PAH substrate binding and/or catalysis. This study expands our understanding of downstream degradation mechanisms for those carcinogenic HMW-PAH pollutants.

## MATERIALS AND METHODS

### Cloning, expression, and purification

The *pahB* gene from strain H2 was amplified and inserted into the pET-28a(+) vector, and the resulting construct was transformed into *E. coli* BL21(DE3), generating the expression strain BL21(PahB). The primers used for plasmid construction are listed in Table S1. For whole-cell biotransformation assays, the RHO-*pahAa/Ab* operon together with *pahB* was assembled into pET-28a(+), whereas RHO-*pahAc/Ad* was introduced into pACYC-Duet1. The two plasmids were co-transformed into *E. coli* BL21(DE3), producing the recombinant strain BL21(PahA + PahB).

For protein production, BL21(pahB) cells were grown in LB medium containing 50 μg/mL kanamycin at 37°C until the OD₆₀₀ reached 0.6. Protein expression was induced with 0.15 mM IPTG at 16°C for 18–20 h. Cells were harvested and resuspended in buffer A (20 mM Tris-HCl, 500 mM NaCl, 20 mM imidazole, pH 8.0), disrupted by sonication, and clarified by centrifugation. The supernatant was loaded onto a Ni²^+^-chelating column, and bound proteins were eluted with buffer A supplemented with 250 mM imidazole. Further purification was performed using a Superdex 75 increase size-exclusion column (GE Healthcare) equilibrated with 50 mM Tris-HCl and 150 mM NaCl (pH 8.0). Protein concentrations were determined by the BCA method, and purified samples were stored at −80°C for subsequent analyses.

### Substrate preparation

Fluoranthene-7,8-dihydrodiol, phenanthrene-3,4-dihydrodiol, and biphenyl dihydrodiol were generated through whole-cell biotransformation using *E. coli* expressing RHO PahA, following the method described previously (19). The resulting dihydrodiol products were subsequently purified and quantified for use in enzymatic assays.

### Whole-cell biotransformation

Whole-cell biotransformation was performed using *E. coli* BL21(PahA + PahB) harboring both *pahA* and *pahB* genes, as previously described (19). The M9 medium was supplemented with 1 mM ascorbic acid to prevent oxidative degradation of catecholic intermediates. Following 48 h of incubation at 25°C with shaking (150 rpm), metabolites were extracted twice with equal volumes of ethyl acetate. The combined organic phases were concentrated by rotary evaporation and redissolved in acetonitrile (18). A portion of the acetonitrile extract was analyzed directly by HPLC, following the previously described protocol (19). The remaining portion was derivatized with a 99:1 mixture of BSTFA and TMCS prior to GC–MS analysis, as described previously (20).

### Steady-state kinetics

Steady-state kinetic parameters of PahB were determined at ambient temperature by monitoring substrate depletion using high-performance liquid chromatography (HPLC). Reaction mixtures (200 μL) contained 50 mM Tris-HCl (pH 8.0), 150 mM NaCl, 1 mM NAD^+^, 20 nM purified PahB, and substrate at various concentrations. Reactions were initiated by addition of the enzyme. For assays with low substrate concentrations, reactions were quenched within 1 min by adding an equal volume of acetonitrile. For higher substrate concentrations, aliquots were withdrawn at 1, 2, 4, and 10 min and quenched in the same manner. Quenched samples were centrifuged and filtered through 0.45-μm membranes, and residual fluoranthene- or phenanthrene-dihydrodiols were quantified by HPLC. Kinetic parameters were obtained by nonlinear regression fitting to the Michaelis–Menten equation (*v* = *V*_max_·[S]/(*K*_m_ + [S])) using Origin. The catalytic constant *k*_cat_ was calculated from *V*_max_ and the total enzyme concentration.

### Sequence alignment and phylogenetic analysis of PahB homologs

Homologous proteins of PahB were identified using NCBI BLASTp (https://blast.ncbi.nlm.nih.gov/) against the Swiss-Prot/PDB database. Functionally characterized homologs were retrieved from literature, with their species origin, functional annotation, and PDB/UniProt accession numbers recorded. Multiple sequence alignment was performed using ClustalW in MEGA11, followed by phylogenetic tree construction with the neighbor-joining (NJ) method (Bootstrap = 1,000). The alignment file (in .aln format) was subsequently visualized using ESPript 3.0 (http://espript.ibcp.fr), with fully conserved residues highlighted in red (100% identity). For conservation analysis of functional motifs, the aligned sequences were processed using WebLogo (https://weblogo.berkeley.edu/) to generate sequence logos illustrating the conservation of key functional residues.

### Crystallization, data collection, and structure determination

Crystals of PahB were obtained by the vapor-diffusion method. Purified PahB was concentrated to 30 mg/mL in 50 mM Tris-HCl and 100 mM NaCl (pH 8.0), and for co-crystallization the protein was pre-incubated with 1 mM NAD^+^ at 4°C for 1 h before crystallization trials. Initial screening was carried out at 25°C using commercial kits from Hampton Research, yielding crystals under conditions containing 0.05 M MgCl₂, 0.1 M HEPES (pH 7.5), and 30% PEG MME 550, or 0.2 M NH₄Cl and 20% PEG 3350. These conditions were further optimized by the hanging-drop vapor-diffusion method to obtain diffraction-quality crystals. For ternary complex preparation, crystals were briefly soaked in reservoir solution supplemented with fluoranthene-dihydrodiol, followed by cryoprotection in reservoir solution containing 15% glycerol and flash-cooling in liquid nitrogen. X-ray diffraction data were collected at beamline BL18U1 of the Shanghai Synchrotron Radiation Facility. Data were processed using XDS and scaled with Aimless. The structure was solved by molecular replacement in PHENIX using an AlphaFold-predicted model as the search template. Iterative model building and refinement were performed with AutoBuild, Coot, and PHENIX. Data collection and refinement statistics are summarized in Table S3.

### Site-directed mutagenesis

Site-directed mutagenesis was performed to generate four PahB variants (M95S, M206I, M209V, and M219V), in which each methionine residue was substituted with the corresponding amino acid found at the equivalent positions of BphB-B-356 (PDB: 3ZV5). Mutagenesis was carried out using a commercial fast mutagenesis kit (Beijing Quanshijin Biotechnology Co., Ltd., China). Mutagenic primers were designed following the manufacturer’s instructions, and the oligonucleotide primers used for site-directed mutagenesis are listed in Table S2. All mutant constructs were sequence-verified and expressed and purified in the same manner as the wild-type PahB.

### Molecular docking

Molecular docking was performed using AutoDock Vina to examine binding modes of four PAH dihydrodiols: phenanthrene-3,4-dihydrodiol, fluoranthene-7,8-dihydrodiol, pyrene-1,2-dihydrodiol, and benzo[a]pyrene-9,10-dihydrodiol. Ligands were generated in ChemDraw 2D, converted to 3D in Chem3D, and energy-minimized. Protein structures were prepared using AutoDockTools by removing water molecules, adding polar hydrogens, and assigning Gasteiger charges, with all preparations visually checked in PyMOL. Docking was performed using a grid box centered at the predicted substrate-binding pocket (center: −6.2, −21.5, −4.5; size: 23.7 × 17.0 × 13.0 Å). For pyrene-1,2-dihydrodiol and benzo[a]pyrene-9,10-dihydrodiol, residue M219 was treated as a flexible side chain to avoid steric hindrance observed in rigid docking.

## RESULTS

### Identification of PahB in strain H2

In our previous study on *Altererythrobacter* sp. H2 (19), a horizontally transferred H2-PAH gene cluster was identified based on the genomic region surrounding the Rieske-type ring-hydroxylating oxygenase genes involved in PAH oxidation. To search for the downstream dihydrodiol dehydrogenase in this pathway, amino acid sequences of functionally characterized PAH-related dihydrodiol dehydrogenases, including classical NahB/BphB-type enzymes, were used to query the H2 genome for homologous proteins. Among the genes within the H2-PAH cluster, one candidate gene showed clear homology to these reported dehydrogenases. Further annotation of this gene cluster against the Swiss-Prot database identified this gene, designated *pahB*, as encoding a putative dihydrodiol dehydrogenase acting downstream of the initial dioxygenation step. Because dihydrodiol dehydrogenases are required to convert cis-dihydrodiol intermediates into catechols for subsequent ring cleavage, PahB was selected for further biochemical and structural characterization. In addition, PahB showed high sequence similarity to BphB-CHY-1, a functionally characterized PAH dihydrodiol dehydrogenase from *Sphingomonas* sp. CHY-1, further supporting its candidacy for processing HMW-PAH-derived dihydrodiols.

**Fig 1:**
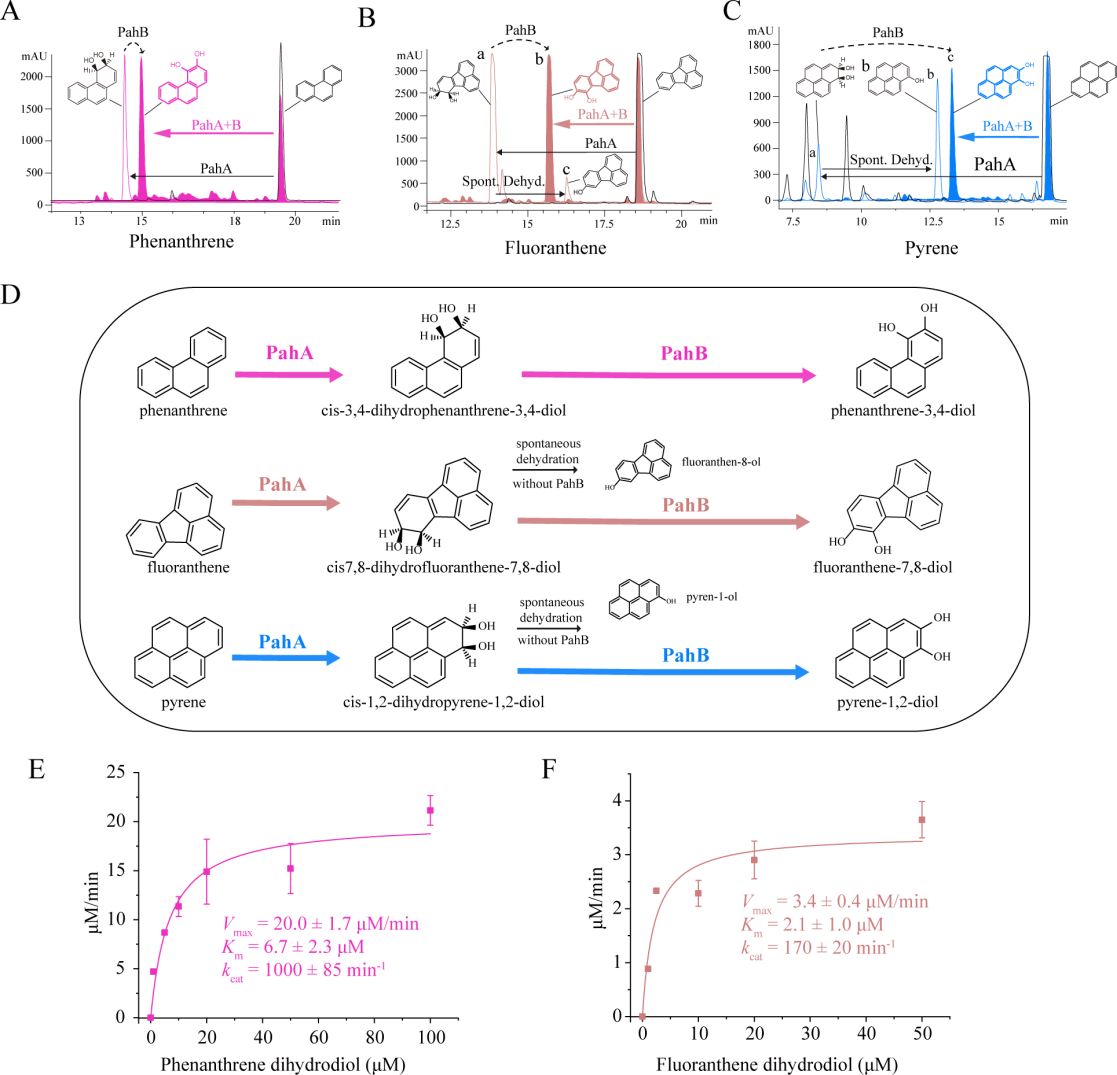
Enzymatic characterization of PahB. (**A–C**) HPLC chromatograms showing the conversion of phenanthrene (**A**), fluoranthene (**B**), and pyrene (**C**) to the corresponding dihydrodiol- and catechol-type products by *E. coli* cells harboring PahA (hollow-colored peaks), PahAB (solid-colored peaks), or the empty vector control (black). Colored, black, and dashed arrows indicate conversions mediated by PahAB, PahA, and the proposed activity of PahB, respectively. Spontaneous dehydration products are labeled where applicable. All reactions were performed in whole-cell systems. Representative HPLC chromatograms are shown. Experiments were performed with three independent biological replicates. (**D**) Proposed biochemical pathway showing the sequential oxidation of phenanthrene, fluoranthene, and pyrene by PahA and PahB. PahA catalyzes the formation of cis-dihydrodiol intermediates, which are further oxidized by PahB to the corresponding dihydroxylated products. Spontaneous dehydration of dihydrodiols leads to mono-hydroxylated byproducts. (**E and F**) Michaelis–Menten kinetics assays of PahB toward phenanthrene dihydrodiol (**E**) and fluoranthene dihydrodiol (**F**). Data points represent mean values from three independent biological replicates, and error bars indicate standard deviations. The fitted kinetic parameters (*V*_max_, *K*_m_, and *k*_cat_) are shown in each panel.

To verify whether PahB indeed functions as the dihydrodiol dehydrogenase in the PAH catabolic pathway of strain H2, we first examined its substrate scope using engineered *E. coli* cells harboring both Rieske-type non-heme iron aromatic ring-hydroxylating oxygenase (RHO) PahA and dihydrodiol dehydrogenase PahB. Because cis-dihydrodiols of polycyclic aromatic hydrocarbons are difficult to prepare directly, we used this whole-cell system to investigate the potential substrates and products of PahB. The productions of phenanthrene 3,4-dihydrodiol, fluoranthene 7,8-dihydrodiol, and pyrene 1,2-dihyrodiol, benzo[a]pyrene-9,10-dihydrodiol were observed in HPLC assays after those *E. coli* cells harboring PahA were individually incubated with phenanthrene (PHE), fluoranthene (FLA), pyrene (PYR), and benzo[a]pyrene (BaP), respectively (19). In contrast, 3,4-dihydroxy-phenanthrene (Phe-3,4-diol), 7,8-dihydroxy-fluoranthene (Fla-7,8-diol), and 1,2-dihydroxy-pyrene (Pyr-1,2-diol) were observed in HPLC assays after those *E. coli* cells harboring both PahA and PahB were incubated with phenanthrene, pyrene, and fluoranthene, respectively (Fig. 1A through C). The production of 9,10-dihydroxy-benzo[a]pyrene was observed for cells harboring PahAB were incubated with BaP only in GC-MS assays, but not in HPLC assays, suggesting very low production of 9,10-dihydroxy-benzo[a]pyrene. Similarly, for some other PAH substrates, including naphthalene and benzo[a]anthracene, no obvious differential peaks were observed in HPLC assays, and the corresponding products were therefore confirmed by GC-MS analysis (Fig. S1). These results indicated that PahB converted phenanthrene 3,4-dihydrodiol, fluoranthene 7,8-dihydrodiol, and pyrene 1,2-dihyrodiol, benzo[a]pyrene-9,10-dihydrodiol to 3,4-dihydroxy-phenanthrene, 7,8-dihydroxy-fluoranthene, 1,2-dihydroxy-pyrene, and 9,10-dihydroxy-benzo[a]pyrene, respectively. Limited mono-hydroxylation products were observed for PahA acting on fluoranthene and pyrene, whereas not for PahAB. This confirmed that those mono-hydroxylation products of RHOs acting on PAHs should be the spontaneous dehydration products of those corresponding diols, not the direct products. Based on these results, a biochemical pathway for the sequential oxidation of PAHs by PahA and PahB is proposed (Fig. 1D).

**Fig 2:**
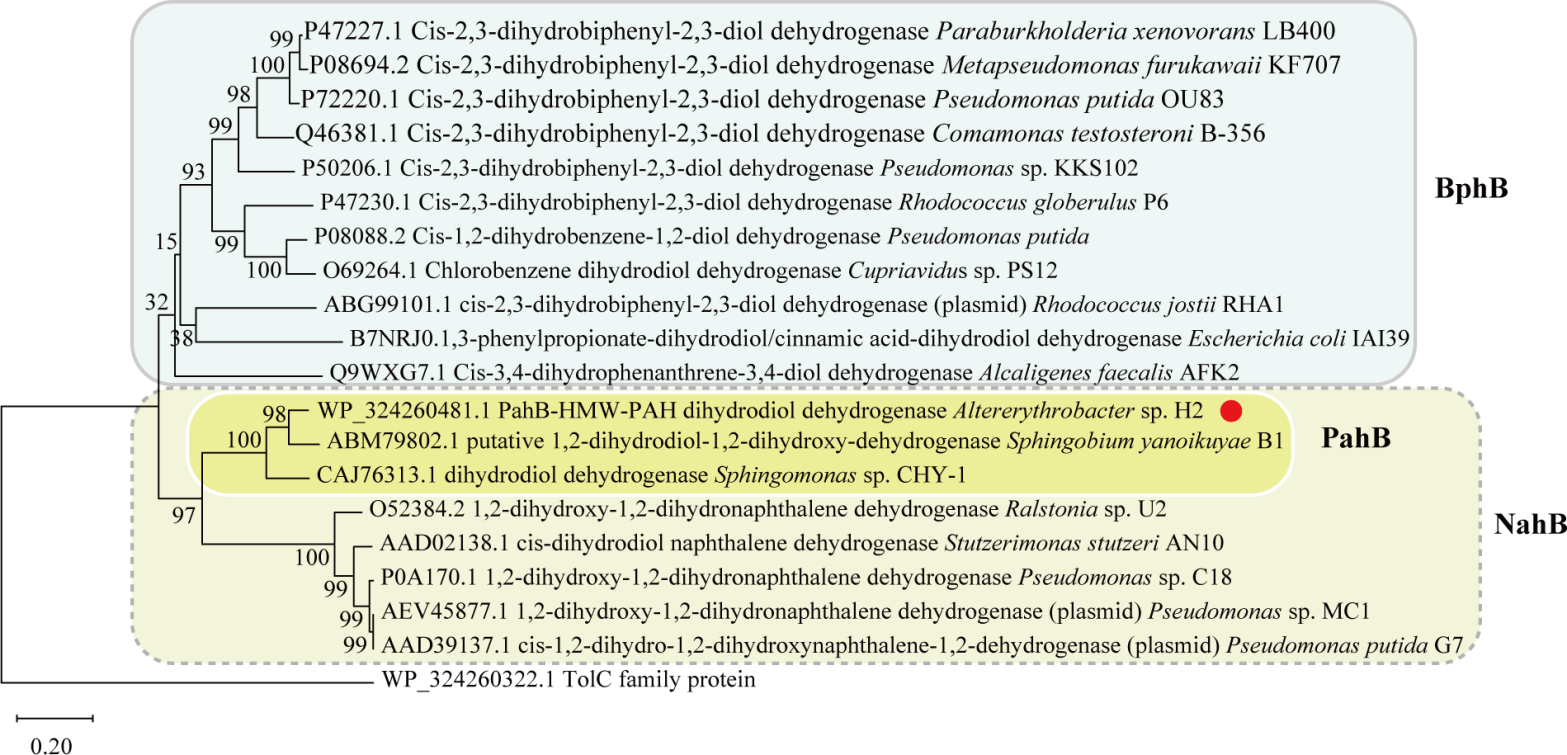
Phylogenetic analysis of PahB. Neighbor-joining phylogenetic tree constructed using amino acid sequences of PahB and other functionally reported cis-2,3-dihydrodiol dehydrogenases. PahB from *Altererythrobacter* sp. H2 is indicated by a red dot. Enzymes are grouped into two major clades, namely the BphB-type clade and the NahB-type clade. PahB clusters within an HMW-PAH-associated subbranch of the NahB-type clade. WP_324260322.1 was used as the outgroup. Bootstrap values (%) based on 1,000 replicates are shown at branch nodes.

The enzymatic kinetics were then assayed using phenanthrene 3,4-dihydrodiol and fluoranthene 7,8-dihydrodiol as substrates. The oxidation of phenanthrene 3,4-dihydrodiol by PahB followed Michaelis–Menten kinetics, with *V*_max_ of 20.0 ± 1.7 µM/min, *K*_m_ of 6.7 ± 2.3 µM, and *k*_cat_ of 1,000 ± 85 min⁻¹ (16.7 ± 1.4 s⁻¹) (Fig. 1E), so did the oxidation of fluoranthene 7,8-dihydrodiol, with *k*_cat_ of 170 ± 20 min⁻¹ (2.8 ± 0.3 s⁻¹), *V*_max_ of 3.4 ± 0.4 µM/min, and *K*_m_ of 2.1 ± 1.0 µM (Fig. 2). These results indicated that PahB exhibited higher substrate affinity toward fluoranthene 7,8-dihydrodiol but a faster turnover rate for phenanthrene 3,4-dihydrodiol. The catalytic efficiency of PahB toward PHE and FLA was comparable to those reported for HMW-PAH-degrading dehydrogenase BphB-CHY-1 from *Sphingomonas* sp. CHY-1, an enzyme with demonstrated HMW-PAH-diol dehydrogenase activity (18).

### Location of PahB in the phylogenetic tree

We next examined the evolutionary relationship and similarity between PahB and previously characterized dihydrodiol dehydrogenases. PahB shared 78.79% identity with BphB-CHY-1 (AM230449.1) from *Sphingomonas* sp. CHY-1. In contrast, it exhibited only moderate similarity with those reported naphthalene-1,2-dihydrodiol (NAP-diol) dehydrogenase NahB (cis-1,2-dihydroxy-1,2-dihydronaphthalene dehydrogenase) and BphB (cis-biphenyl dihydrodiol dehydrogenase) orthologs from other genera, including NahB (P0A169.1) from *Pseudomonas putida* BS202 (47.27%), NagB (O52384.2 from *Ralstonia* sp. U2 (50.78%), NagB (Q46381.1) from *Comamonas testosteroni* B-356 (44.75%), and BphB (P47230.1) from *Rhodococcus globerulus* P6 (43.80%). Phylogenetic analyses indicate that PahB is most closely related to NahB-type PAH-associated dihydrodiol dehydrogenases rather than to biphenyl-type BphB homologs. In the phylogenetic tree (Fig. 2), PahB clustered with the HMW-PAH-degrading dehydrogenase BphB-CHY-1 from *Sphingomonas* sp. CHY-1 and was positioned close to other NahB/NagB-related enzymes, while remaining distinct from the branch formed by biphenyl-associated BphB homologs. These results suggest that PahB belongs to a PAH-associated subgroup within the NahB-type branch, and that PahB and BphB-CHY-1 are associated with preference toward HMW-PAH-derived substrates.

### Crystal structure of PahB

**Fig 3:**
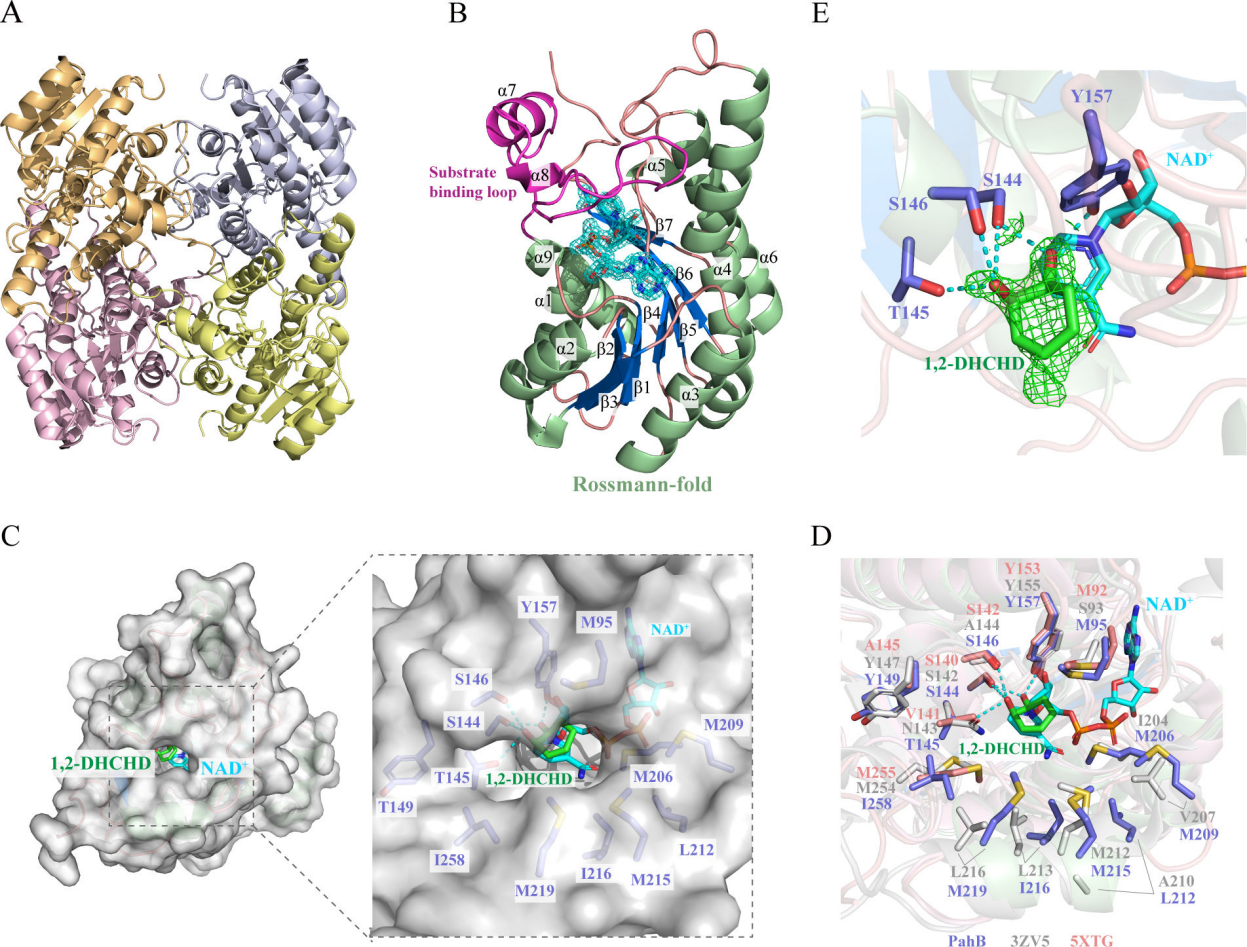
Overall structure of PahB and its substrate-binding pocket. (**A**) Overall structure of PahB tetramer, with the four subunits shown in different colors. (**B**) Cartoon representation of a PahB monomer. β-strands, α-helices, and the substrate-binding loop are colored dark blue, green, and magenta, respectively, and secondary-structure elements are labeled sequentially. (**C**) The potential substrate-binding pocket and the modeled surrogate ligand. The cavity accommodating 1,2-DHCHD and NAD^+^ is shown on the left, with a magnified inset highlighting pocket-forming residues, M95, M206, M209, L212, I216, M219, and I258, in stick representation. (**D**) Structural comparison of PahB with related homologs (PDB IDs: 3ZV5 and 5XTG) highlighting the substrate-binding pocket region. PahB is shown in slate, 3ZV5 in gray, and 5XTG in salmon. Notably, 5XTG lacks a segment near the substrate-binding pocket (5XTG: L187–T216), which is absent in the corresponding region of PahB and 3ZV5. (**E**) Fo–Fc ligand-omit electron-density map (green mesh) in the substrate-binding region. The density was modeled using 1,2-DHCHD as a structural surrogate for the dihydrodiol moiety.

To elucidate the structural basis of the PahB dehydrogenase activity, the crystal structure of PahB in complex with NAD^+^ was first determined at resolution of 1.6 Å. Only one PahB molecule was observed in the asymmetric unit. However, it formed a tetramer through crystallographic symmetry (Fig. 3A), in agreement with the oligomeric state observed by size-exclusion chromatography (Fig. S2). The structure adopted the canonical Rossmann-fold core characteristic of short-chain dehydrogenase/reductase (SDR) enzymes, which span residues M1–G186 and P228–S254 and was inserted by a potential substrate-binding loop (G187–E227) (Fig. 3B). In detail, a β-sheet was formed by β-strands β1 (V9–T13), β2 (K33–V38), β3 (V55–A59), β4 (V84–G87), β5 (S138–T142), β6 (R180–P186), and β7 (V251–S254), and flanked by α-helices α1 (G18–E29), α2 (D41–S54), and α9 (P228–A239) from one side and by α3 (Y65–F79), α4 (P103–V135), α5 (T145–S147), and α6 (T155–L175) from the other side, and helices α7 (M206–Q208) and α8 (L212–M219) located at the substrate-binding loop that extended above the β-sheet.

The overall structure was highly similar to previously reported BphB from *Pandoraea pnomenusa* strain B-356 (PDB 3ZV5 [21]) and NahB from *Pseudomonas* sp. MC1 (PDB 5XTG [17]), with Cα r.m.s.d of 2.0 and 2.1 Å, respectively. A NAD^+^ molecule was located over the β-sheet and was buried by the potential substrate-binding loop. An ethylene glycol molecule (EG) clamped by the nicotinamide ring of NAD^+^ from one side and by the indole ring of residue W92 from the other side was considered as the potential substrate binding site (Fig. S3A). Structural superposition of PahB with NahB (PDB 5XTG) and BphB (PDB 3ZV5, 3ZV6) showed a nearly identical cofactor-binding architecture, suggesting a common cofactor binding mode (Fig. S2B and C).

We next determined the structure of PahB crystal after soaking with fluoranthene-2,3-dihydrodiol. Unambiguous electron density was observed near NAD^+^, but this density could not be assigned to the full fluoranthene dihydrodiol molecule. Instead, the clearest interpretable density corresponded to a single aromatic ring with the adjacent dihydrodiol moiety in the substrate-binding region, whereas the remaining polycyclic portion was not sufficiently resolved, likely due to partial occupancy and/or conformational disorder of the bulky ligand. To represent this density in a chemically reasonable manner, we modeled it using (1R,2S)-cyclohexa-3,5-diene-1,2-diol (1,2-DHCHD) as a structural surrogate for the dihydrodiol moiety, rather than as a physiological substrate of PahB (Fig. S3A). The substrate-binding pocket is methionine-rich and hydrophobic, and is formed by residues Y149, Y157, L212, I216, I258, M95, M206, M209, M215, and M219 (Fig. 3C and D). These methionine residues are located around the entrance of the substrate-binding pocket, where their side chains may provide a hydrophobic environment for ligand access (Fig. 3D). Comparison with 5XTG revealed a deletion in the substrate-entry region, whereas comparison with 3ZV5 showed substantial differences in amino-acid composition within the substrate-binding pocket (Fig. 3D). In this model, the six-membered ring is clamped by the nicotinamide ring of NAD^+^ on one side and by the indole ring of W92 on the other side. One hydroxyl group interacts with T145, S146, and T150, and the other with S146 and Y157 (Fig. 3E).

### Docking of PAH dihydrodiols to PahB

**Fig 4:**
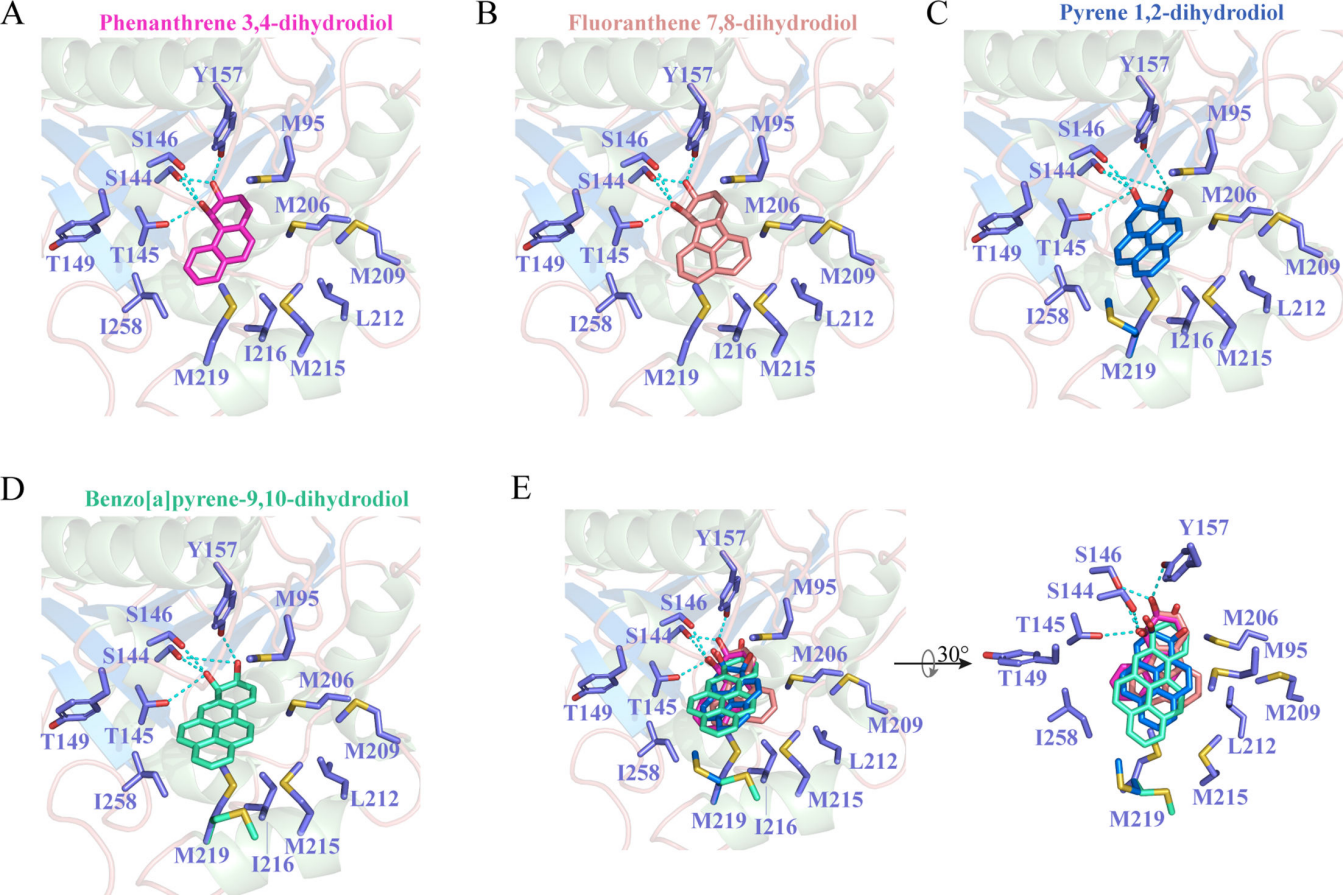
Docking assays of different PAH dihydrodiols into the active site of PahB. (**A–D**) Docking poses of phenanthrene-3,4-dihydrodiol (**A**), fluoranthene-7,8-dihydrodiol (**B**), pyrene-1,2-dihydrodiol (**C**), and benzo[a]pyrene-9,10-dihydrodiol (**D**) within the substrate-binding pocket of PahB. Each substrate is shown in a distinct color. The vicinal diol groups form conserved hydrogen bonds with S144, T145, S146, and Y157, while the aromatic rings extend into the hydrophobic pocket formed by M95, M206, M209, L212, I216, M219, and I258. (**E**) Superposition of the four docked PAH dihydrodiols in the substrate-binding pocket of PahB. A front view on the right is obtained by rotating the model by 30°. Three conformations of M219 are shown: the crystallographic conformation (purple), a rotamer sampled in the pyrene-dihydrodiol docking model (blue), and a distinct rotamer in the benzo[a]pyrene-dihydrodiol docking model (green).

To further elucidate the structural basis for substrate recognition and the accommodation of HMW-PAHs by PahB, molecular docking analyses were performed using representative PAH dihydrodiols as ligands. Docking results indicated that phenanthrene-3,4-dihydrodiol, fluoranthene-7,8-dihydrodiol, pyrene-1,2-dihydrodiol, and benzo[a]pyrene-9,10-dihydrodiol can be accommodated within the same binding pocket of PahB (Fig. 4A through E), adopting a conserved binding orientation. The two hydroxyl groups of phenanthrene-, pyrene-, fluoranthene-, and benzo[a]pyrene-derived dihydrodiols were positioned similarly to those of the modeled 1,2-DHCHD surrogate in the crystal structure (Fig. 4E). In detail, residues T145, S146, and T150 formed hydrogen bonds with one hydroxyl group, and S146 and Y157 with the other one. This finding indicates that PahB recognizes diverse PAH dihydrodiols through a consistent hydroxyl-group recognition mechanism. Surrounding hydrophobic residues (W92, M95, M206, M209, L212, M219, I216, and I258) further stabilized substrate binding through aromatic interactions. Notably, docking of pyrene-1,2-dihydrodiol and benzo[a]pyrene-9,10-dihydrodiol, but not the other substrates, suggested alternative side-chain conformations of residue M219, supporting a potential role for M219 in accommodating these bulky molecules.

### Roles of methionine residues in subtrate-binding pocket

**Fig 5:**
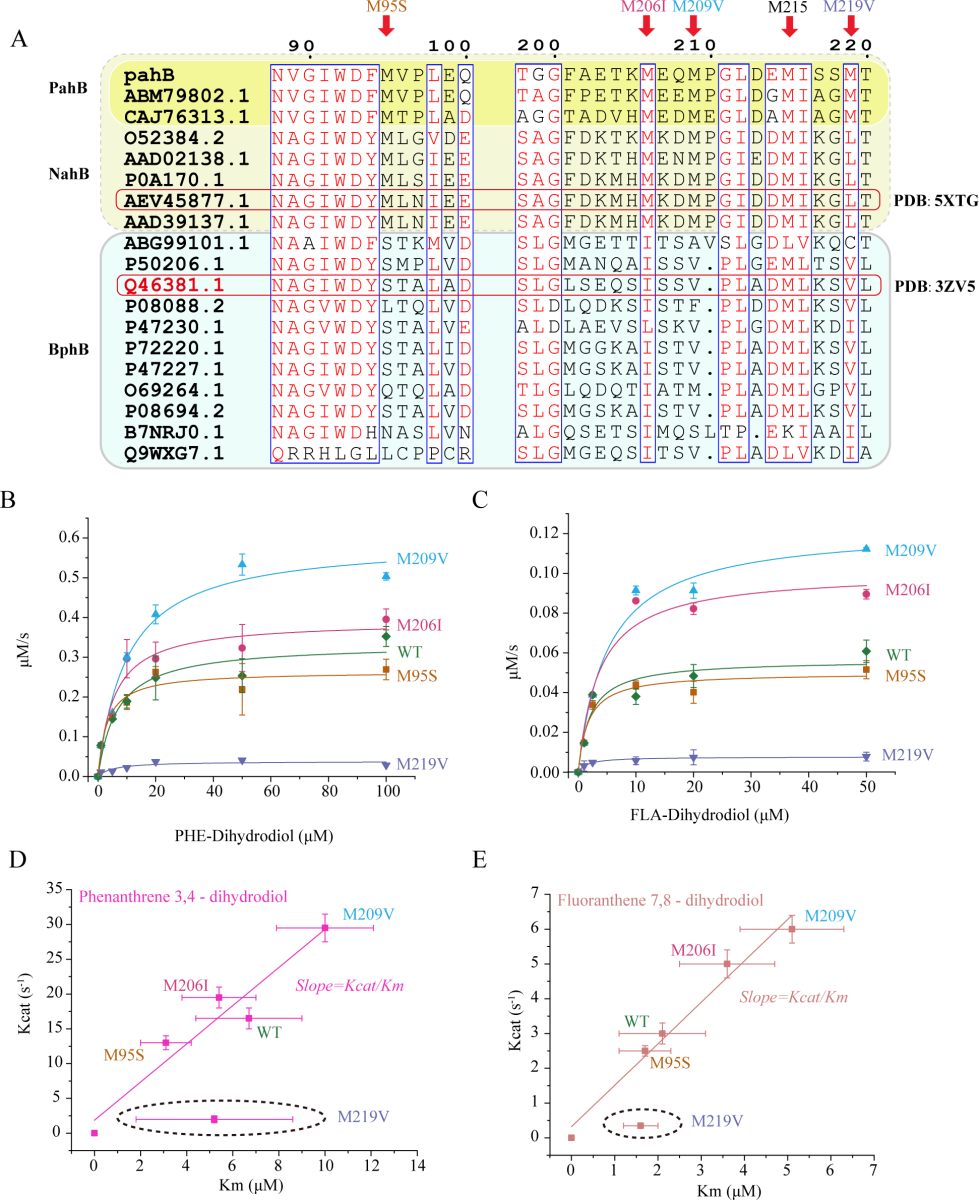
Sequence alignment of PahB similarities and kinetic characterization of PahB mutants. (**A**) Sequence alignment of PahB with representative homologs from the NahB and BphB families. Conserved residues corresponding to M95, M206, M209, M215, and M219 are marked. (**B and C**) Kinetic assays of PahB mutants using phenanthrene-3,4-dihydrodiol (**B**) or fluoranthene-7,8-dihydrodiol (**C**) as substrates. Wild-type PahB is shown in green; M95S in brown; M206I in magenta; M209V in cyan; and M219V in purple. Experiments were performed with three independent biological replicates, and error bars indicate standard deviations. (**D and E**) Plots showing the *k*_cat_ and *K*_m_ values of PahB and mutants toward phenanthrene-3,4-dihydrodiol (**D**) and fluoranthene-7,8-dihydrodiol (**E**). All mutants except M219V could be linearly fitted, indicating that they shared similar catalytic efficiencies (*k*_cat_*/K*_m_).

We observed some methionine residues, M95, M206, M209, M215 and M219, were involved in the potential substrate-binding pocket. Sequence alignment and structural superposition revealed that the equivalent positions are occupied by smaller or less flexible residues—S93, I204, V207, M212, and V216 in cis-biphenyl dihydrodiol dehydrogenase (BphB; PDB 3ZV5). In contrast, only methionine residue, M219, was replaced by leucine in NahB that was involved in LMW-PAH degradation (NahB; PDB 5XTG) (Fig. 5A). The above docking analyses suggested that binding of pyrene- and benzo[a]pyrene-derived dihydrodiols may require alternative side-chain conformations of M219, implicating this residue in accommodation of bulky substrates. To gain insight into the structural basis of how PahB accumulated HMW PAH-diol, we substituted those residues in PahB with the corresponding residues from BphB to get mutants M95S, M206I, M209V, and M219V (Fig. 5A), and then accessed the role of those four residues in enzymatic activities.

For phenanthrene 3,4-dihydrodiol (Fig. 5D), M95S (*K*_m_ of 3.1 ± 1.1 µM, *k*_cat_ of 13.0 ± 1.0 s⁻¹) and 206I (5.4 ± 1.6 µM, 19.5 ± 1.5 s⁻¹) displayed comparable *K*_m_ and *k*_cat_ values with wild-type PahB (6.7 ± 2.3 µM, 16.5 ± 1.5 s⁻¹), M209V (10.0 ± 2.1 µM, 29.5 ± 2.0 s⁻¹) had the much higher *K*_m_ and *k*_cat_ values than wild-type PahB, whereas M219V (5.2 ± 3.4, 2.0 ± 0.5 s⁻¹) nearly lost activity with drastically decreased *k*_cat_ values. Wild-type PahB exhibited a catalytic efficiency (*k*_cat_ /*K*_m_) of 2.46 ± 1.2 µM⁻¹ s⁻¹. The M209V, M206I, and M95S variants showed a little higher, but comparable, efficiencies (2.95 ± 1.1, 3.61 ± 1.4, and 4.19 ± 1.6 µM⁻¹ s⁻¹, respectively), whereas M219V displayed a significantly lower efficiency (0.38 ± 0.25 µM⁻¹ s⁻¹) (Fig. 5B).

For fluoranthene 7,8-dihydrodiol (Fig. 5E), M206I (3.6 ± 1.1 µM, 5.0 ± 0.4 s⁻¹) and M209V (5.1 ± 1.2 µM, 6.0 ± 0.4 s⁻¹) maintained a little higher *K*_m_ and *k*_cat_ values than wild-type PahB (2.1 ± 1.0 µM, 3.0 ± 0.3 s⁻¹). In contrast, M95S (1.7 ± 0.6 µM, 2.5 ± 0.15 s⁻¹) showed a little lower *K*_m_ and *k*_cat_ values than wild-type PahB. M219V (1.6 ± 0.4 µM, 0.35 ± 0.02 s⁻¹) exhibited drastically reduced *k*_cat_, indicating markedly impaired catalytic activity. Wild-type PahB showed a catalytic efficiency (*k*_cat_*/K*_m_) of 1.43 ± 0.7 µM⁻¹ s⁻¹ for fluoranthene 7,8-dihydrodiol. The M95S, M206I, and M209V mutants maintained comparable catalytic efficiencies (1.47 ± 0.6 µM⁻¹ s⁻¹, 1.39 ± 0.5, and 1.18 ± 0.4 µM⁻¹ s⁻¹, respectively), whereas M219V exhibited a significant decrease in catalytic efficiency (0.22 ± 0.07 µM⁻¹ s⁻¹) (Fig. 5C).

For biphenyl dihydrodiol (Fig. S4A), M206I (5.2 ± 2.1 µM, 11.0 ± 1.0 s⁻¹) exhibited markedly increased *K*_m_ and *k*_cat_ values relative to wild-type PahB (1.5 ± 0.4 µM, 3.0 ± 0.1 s⁻¹). M219V (5.8 ± 0.9 µM, 4.5 ± 0.15 s⁻¹) also showed increased *K*_m_ and *k*_cat_ values compared with the wild type. By contrast, M209V (2.0 ± 0.8 µM, 3.0 ± 0.2 s⁻¹) displayed a slightly increased *K*_m_ while maintaining a *k*_cat_ similar to that of wild-type PahB. In contrast, M95S (0.6 ± 0.3 µM, 1.0 ± 0.05 s⁻¹) showed the lowest *K*_m_ and *k*_cat_ values among the tested variants. Wild-type PahB exhibited a catalytic efficiency (*k*_cat_/*K*_m_) of 1.19 ± 0.46 µM⁻¹ s⁻¹ toward biphenyl dihydrodiol. The M206I and M209V variants showed overall comparable catalytic efficiencies (2.12 ± 0.88 and 1.50 ± 0.61 µM⁻¹ s⁻¹, respectively), whereas M219V displayed a moderately reduced efficiency (0.78 ± 0.12 µM⁻¹ s⁻¹) (Fig. S4B). Although M95S yielded an apparently higher *k*_cat_/*K*_m_ value (1.67 ± 0.84 µM⁻¹ s⁻¹), the large associated error suggests high variability and prevents a firm conclusion that catalytic efficiency was improved. Overall, unlike the pattern observed for HMW-PAH-derived substrates, M219V did not exhibit a pronounced loss of catalytic efficiency toward biphenyl dihydrodiol, suggesting that the effects of methionine substitutions are substrate dependent.

These results indicate that methionine residues nearby the active pocket contribute differentially to substrate accommodation. Residues M95, M206, and M209 primarily fine-tune substrate affinity and turnover, resulting in relatively stable catalytic efficiencies through a trade-off between *K*_m_ and *k*_cat_. In contrast, residue M219 plays a distinct role in accommodating bulky HMW-PAH-derived substrates, as its substitution markedly impaired catalytic efficiency toward phenanthrene and fluoranthene dihydrodiols but had a much smaller effect on biphenyl dihydrodiol. Consistent with this observation, M219 is not conserved in cis-biphenyl dihydrodiol dehydrogenases and naphthalene dihydrodiol dehydrogenases, suggesting that this residue contributes specifically to the adaptation of PahB toward HMW-PAH substrates rather than being required for catalysis of smaller substrates.

## DISCUSSION

This work provides the first structural insight into a dihydrodiol dehydrogenase that specifically acts on high-molecular-weight PAH intermediates. The crystal structures of PahB, together with functional and mutational analyses, reveal the unique structural features that enable efficient oxidation of four- to five-ring PAH dihydrodiols. These findings establish PahB as a representative of a previously uncharacterized class of dehydrogenases specialized for bulky PAH substrates, thereby advancing our understanding of the downstream steps in HMW-PAH biodegradation.

Co-expression of PahA and PahB enabled efficient oxidation of four- to five-ring PAHs, including benzo[a]pyrene, pyrene and fluoranthene, confirming their cooperative role in the catabolism of high-ring PAHs. Traditionally, PahA alone has been used for such experiments; however, when PahA was expressed independently, the major products were monohydroxylated intermediates, such as pyrene monohydroxide. For fluoranthene, both mono- and dihydroxylated derivatives were detected. We propose that these apparent monohydroxylated products likely arise from the spontaneous dehydration of unstable cis-dihydrodiol intermediates formed by PahA in the absence of PahB. By co-expressing PahB with PahA in the same *Escherichia coli* strain, these side products were effectively eliminated, yielding only a single catechol-like metabolite. This approach overcomes the issue of nonspecific hydroxylation or spontaneous dehydration of hydroxylated products by PahA alone, providing a more accurate method for studying the specific hydroxylation sites of PahA.

Phylogenetic and structural analyses suggest that PAH dihydrodiol dehydrogenases can be divided into two major functional classes with distinct substrate preferences: biphenyl-type enzymes with compact aliphatic pockets, and NahB-type PAH-associated enzymes that retain several methionines. Within the latter class, PahB and the HMW-PAH-degrading BphB-CHY-1 from *Sphingomonas* CHY-1 form an HMW-PAH-associated subbranch. Within the NahB-type PAH-associated, sequence and functional differences further suggest variation in substrate preference between enzymes acting mainly on lower-ring PAHs and those associated with HMW-PAH-derived substrates. Although these enzymes share the canonical SDR Rossmann-fold, PahB and the HMW-PAH-associated homolog BphB-CHY-1 within the NahB-type PAH-associated contain a methionine-rich substrate pocket with greater apparent conformational adaptability than that of biphenyl-type BphB homologs. In naphthalene-type enzymes, the corresponding position is occupied by leucine, whose more compact side chain likely provides less conformational flexibility than the methionine present at the equivalent position in PahB. By contrast, PahB contains a cluster of methionines that shapes a spacious and dynamic hydrophobic cavity capable of accommodating four- and five-ring PAH dihydrodiols.

Docking analyses suggested that alternative side-chain conformations of M219 may help relieve steric clashes with bulky ligands, and mutagenesis further supported an important role for this residue: substitution of M219 with valine drastically reduced catalytic efficiency toward phenanthrene and fluoranthene dihydrodiols but caused only a modest reduction toward biphenyl dihydrodiol. This substrate-dependent pattern indicates that M219 is not generally required for catalysis but may contribute specifically to accommodation of bulky HMW-PAH-derived intermediates. Together, these results support a model in which M219 may act as a flexible hydrophobic gate that facilitates processing of large polycyclic substrates, rather than being required for catalysis of smaller substrates.

Our findings demonstrate that the effective substrate range of a PAH catabolic pathway is not determined solely by the initial RHOs. While RHOs define which PAHs can be initially oxidized, the resulting cis-dihydrodiols cannot enter the ring-cleavage pathway unless they are rapidly converted by dehydrogenase. Despite their functional interdependence, the RHO and dehydrogenase genes in strain H2 are not positioned adjacently within the same genomic region, suggesting that their pairing reflects functional selection rather than strict genomic coevolution. Consequently, evaluating or engineering the PAH substrate range of a microbial system requires considering both enzymatic steps. RHO activity alone does not fully predict whether HMW-PAH-derived intermediates can be productively processed through the downstream pathway. These results do not indicate that dehydrogenase is necessarily rate-limiting, but rather that downstream substrate compatibility must also be considered when evaluating the effective substrate scope of a PAH catabolic system.

Despite these advances, some limitations of the present study should be noted. Because PAH dihydrodiol intermediates, particularly those derived from high-molecular-weight substrates, such as benzo[a]pyrene and pyrene, are scarce and unstable, a systematic *in vitro* evaluation of PahB with all potential substrates was not feasible. In addition, the weak NADH signal generated during fluoranthene dihydrodiol oxidation precluded reliable cofactor-based kinetic analysis, and kinetic characterization for this substrate therefore relied on substrate-depletion measurements. Accordingly, the substrate scope and product formation of PahB were assessed mainly using Escherichia coli whole-cell transformation assays. For some PAH substrates, no clearly distinguishable differential peaks were observed by HPLC, and the corresponding products were therefore confirmed by the more sensitive GC-MS analysis. These analytical differences may limit direct quantitative comparison of product formation across substrates.

In summary, our work identifies M219 as the structural feature that enables PahB, but not classical naphthalene- or biphenyl-type dehydrogenases, to process bulky HMW-PAH dihydrodiols. This finding highlights that PAH substrate specificity arises from the combined selectivities of both the RHO and its partnering dehydrogenase, rather than from the oxygenase alone, offering a clearer framework for predicting and engineering PAH-degrading pathways.

## Data Availability

Two crystal structures of the PAH dihydrodiol dehydrogenase PahB from *Altererythrobacter* sp. H2 generated in this study have been deposited in the Protein Data Bank (http://www.rcsb.org/pdb). The binary complex of PahB with NAD⁺ is available under the accession code 21FN, and the ternary complex containing PahB, NAD⁺, and the substrate is deposited under the accession code 21JM. The *pahB* gene of strain H2 is available in the NCBI database under accession number WRK94617.

## SUPPLEMENTAL MATERIAL

Supplemental material aem.00121-26-s0001.docx Fig. S1 to S4, supplemental results, and Tables S1 to S3.

ASM does not own the copyrights to Supplemental Material that may be linked to, or accessed through, an article. The authors have granted ASM a non-exclusive, world-wide license to publish the Supplemental Material files. Please contact the corresponding author directly for reuse.
