# Supplementary material for: Structural and functional characterization of dihydrodiol dehydrogenase PahB recognizing high-molecular-weight PAH substrates
Source: Appl Environ Microbiol. 2026 Jun 24;92(7):e00121-26. doi: 10.1128/aem.00121-26 (PMC13390466; doi:10.1128/aem.00121-26)
Supplement: Supplemental material — Fig. S1 to S4, supplemental results, and Tables S1 to S3. [file aem.00121-26-s0001.docx]

**Supplementary Material**

**Structural and functional characterization of dihydrodiol dehydrogenase PahB recognizing high-molecular weight-PAHs substrates**

Qun Han^a, b^ ^†^, Lin-Lin Tian^a, b^ ^†^, Lu Guo ^a^, Rui Cui ^a^, Ze-Shen Liu ^a^, De-Feng Li ^a, b^ *

^a^ State Key Laboratory of Microbial Diversity and Innovative Utilization, Institute of Microbiology, Chinese Academy of Sciences, Beijing 100101, China

^b^ College of Life Sciences, University of Chinese Academy of Sciences, Beijing, China.

*Correspondence to: De-Feng Li (lidefeng@im.ac.cn).

† These authors contributed equally to this work.

**This Supplementary Information file includes: Fig. S1-S4, Table S1-S3, and supplementary results.**

Fig. S1


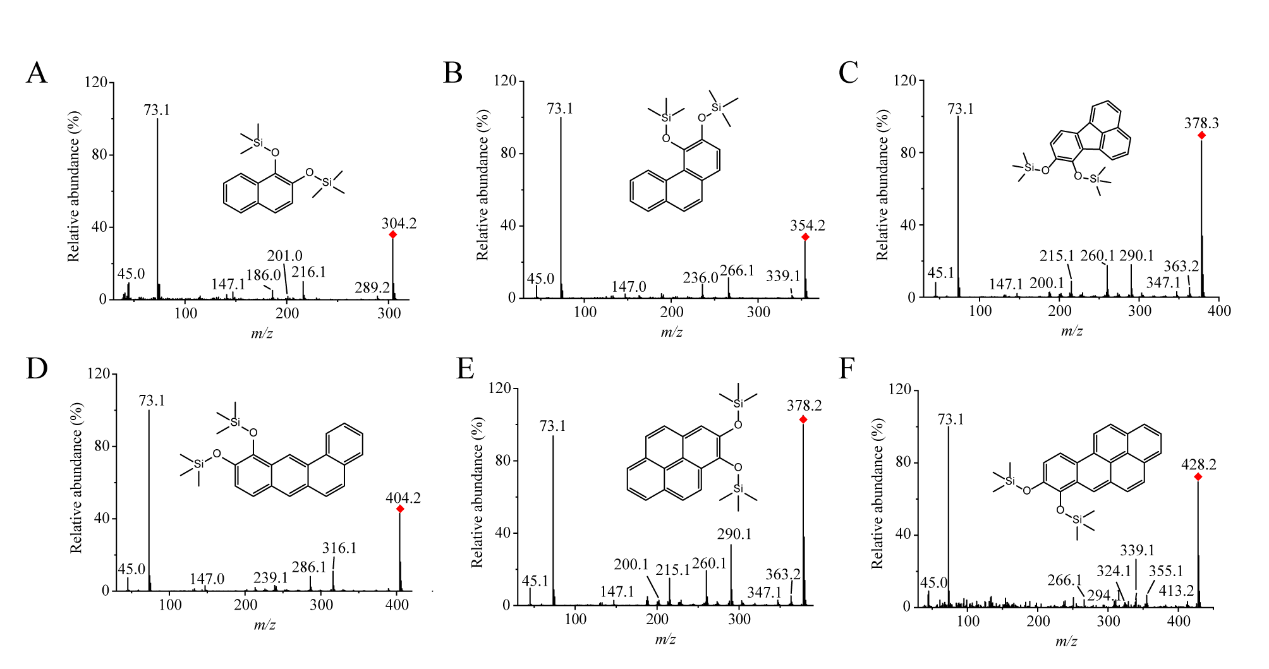


**Fig. S1 GC–MS analysis of trimethylsilylated PAH catechol products generated by PahAB.**

(A) Naphthalene-1,2-diol derivative (RT = 21.12 min), showing characteristic ions at m/z 73.1, 147.1, 186.0, 201.0, 216.1, 289.2, and 304.2. (B) Phenanthrene-3,4-diol derivative (RT = 27.84 min), with major ions at m/z 73.1, 147.0, 256.0, 266.1, 339.1, and 354.2. (C) Pyrene-1,2-diol derivative (RT = 27.84 min), exhibiting predominant ions at m/z 73.1, 147.1, 200.1, 215.1, 260.1, 347.1, 363.2, and 378.2. (D) Fluoranthene-7,8-diol derivative (RT = 31.38 min), showing characteristic ions at m/z 73.1, 147.1, 200.1, 215.1, 260.1, 347.1, 363.2, and 378.3. (E) Benzo[a]anthracene-5,6-diol derivative (RT = 32.98 min), with diagnostic ions at m/z 73.1, 147.0, 239.1, 286.1, 316.1, and 404.2. (F) Benzo[a]pyrene-9,10-diol derivative (RT = 39.05 min), detected with major ions at m/z 73.1, 266.1, 294.1, 324.1, 339.1, 355.1, 413.2, and 428.2.

Fig. S2


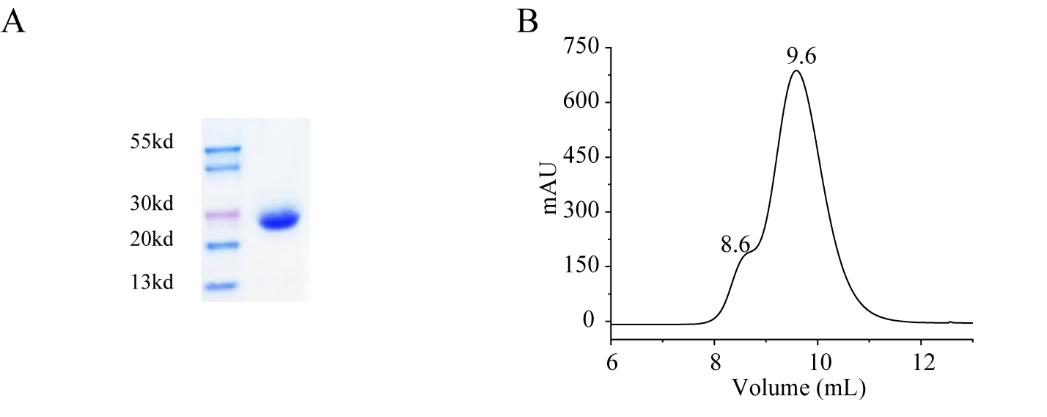


**Fig. S2 Purification and oligomeric state analysis of PahB.**

(A) SDS–PAGE analysis of purified PahB. The observed band migrated between 20 and 30 kDa, consistent with the predicted molecular mass of 27.99 kDa.

(B) Size-exclusion chromatography profile of PahB on a Superdex 75 Increase column. Two major elution peaks were detected at 8.6 mL and 9.6 mL, corresponding to estimated molecular masses of ~154 kDa and ~99 kDa, respectively. The higher molecular weight peak likely represents a soluble oligomeric species, supporting a tetrameric assembly of PahB in solution.

Fig. S3


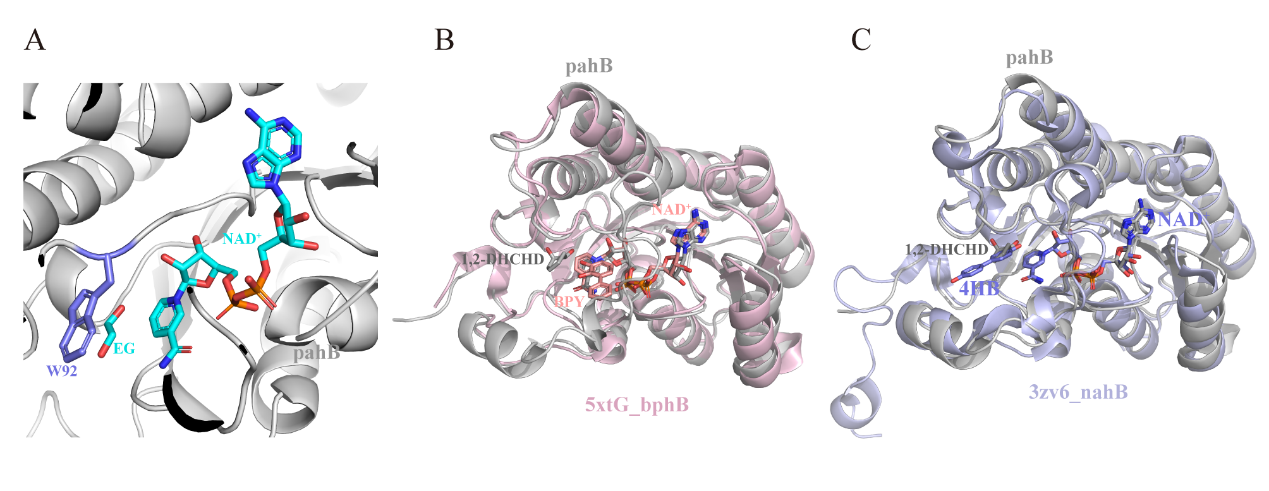


**Fig. S3 Cofactor binding mode of PahB and structural comparison with related dehydrogenases.**

(A) Close-up view of the PahB active site showing NAD⁺ and an ethylene glycol (EG) molecule. The EG molecule is positioned between the nicotinamide moiety of NAD⁺ and the indole ring of Trp92. (B) Structural superposition of PahB with BphB from *Pandoraea pnomenusa* B-356 (PDB: 5XTG). (C) Structural alignment of PahB with NahB from *Pseudomonas* sp. MC1 (PDB: 3ZV6)


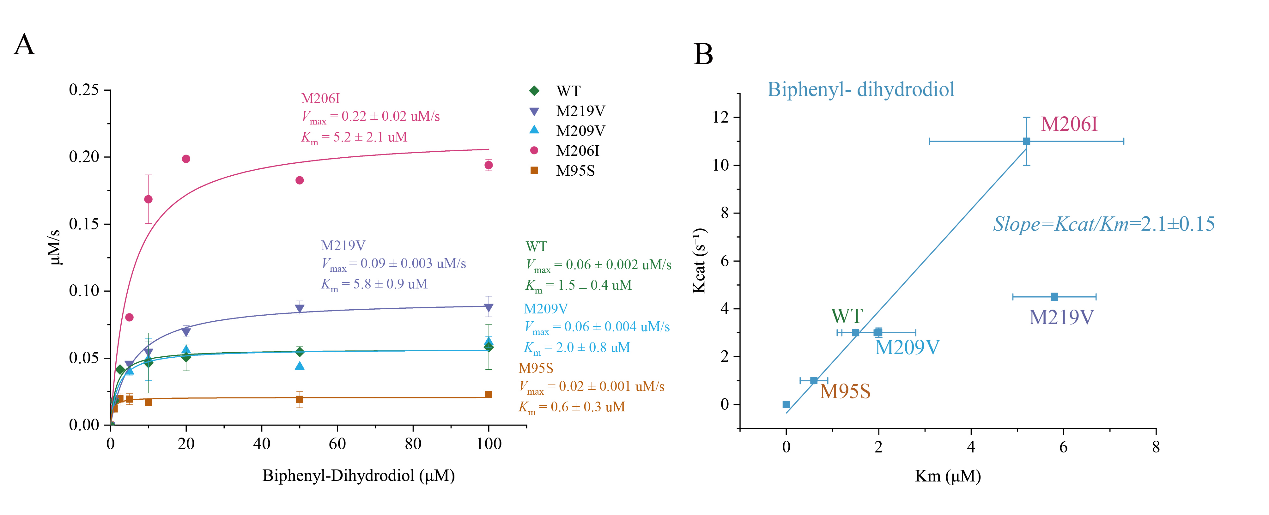


**Fig. S4 Kinetic analysis of PahB mutants using biphenyl dihydrodiol as substrate.**

(A) Michaelis–Menten kinetics of wild-type PahB and the M95S, M206I, M209V, and M219V mutants toward biphenyl dihydrodiol. Initial rates were determined at different substrate concentrations and fitted to the Michaelis–Menten equation. Experiments were performed with three independent biological replicates, and error bars indicate standard deviations. (B) Plot showing the relationship between *k*_cat_ and *K*_m_ values of wild-type PahB and the four mutants toward biphenyl dihydrodiol. The slope from the origin to each point represents catalytic efficiency (*k*_cat_*/K*_m_).

**Supplementary results – Molecular docking analysis**

Molecular docking was performed to evaluate the binding capacities of PahB toward representative PAH dihydrodiols. The predicted binding free energies were −6.7 kcal/mol for phenanthrene-3,4-dihydrodiol, −6.3 kcal/mol for fluoranthene-1,2-dihydrodiol, −7.5 kcal/mol for pyrene-1,2-dihydrodiol, and −8.7 kcal/mol for benzo[a]pyrene-9,10-dihydrodiol.

**Table S1 Primers used for plasmid construction in this study**

| **Primers** | **Primers sequence** | **Plasmids** |
| --- | --- | --- |
| T7-F | TAATACGACTCACTATAGGG | Validation primers |
| T7-R | TGCTAGTTATTGCTCAGCGG | Validation primers |
| PahB-F-1 | GGGAATTCCATATGTCTAACAGGCTTGATGGACAGG | pET28a |
| PahB-R-1 | ACGCGTCGACTCACCCTTCCGGACGTTTGC | pET28a |
| RHO-PahAa/Ab-F | GCCGCGCGGCAGCCATATGATGCCTGGAACCACCAACC | pET28a |
| RHO-PahAa/Ab-R | CGGCTGGTTTAGCAGAAAAAGTACAGGTTCTTGTCTTGC | pET28a |
| RHO-PahAc-F | CTGCTAAACCAGCCGGGCGT | pET28a |
| RHO-PahAc-R | GGGGCGGCTCAGGCAGACTGCTTGAGCAG | pET28a |
| RHO-PahAd-F | TGCCTGAGCCGCCCCCAGAATCG | pET28a |
| RHO-PahAd-R | TGGTGGTGGTGGTGCTCGAGTCAGGCAACTCCGTCTGGTTGA | pET28a |
| PahB-F-2 | CATCACCACAGCCAGGATCCTTGTCTAACAGGCTTGATGGACAGG | pACYC-Duet1 |
| PahB-R-2 | CATTATGCGGCCGCAAGCTTTCACCCTTCCGGACGTTTG | pACYC-Duet1 |

**Table S2 List of oligonucleotide primers for site-specific mutagenesis**

| **Primers** | **Primers sequence** | **Plasmids** |
| --- | --- | --- |
| 95-M-F | GGATCTGGGATTTTAGTGTCCCGCTCG | pET28a |
| 95-M-R | ACTAAAATCCCAGATCCCAACGTTCCCG | pET28a |
| 206-M-F | TTGCAGAGACGAAGATAGAACAGATGCCAG | pET28a |
| 206-M-R | TATCTTCGTCTCTGCAAAGCCACCCGT | pET28a |
| 209-M-F | GACGAAGATGGAACAGGTGCCAGGGCT | pET28a |
| 209-M-R | CCTGTTCCATCTTCGTCTCTGCAAAGCCACC | pET28a |
| 219-M-F | GAAATGATTTCCAGCGTGACCCCGCTCGG | pET28a |
| 219-M-R | CGCTGGAAATCATTTCGTCAAGCCCTGGC | pET28a |

**Table S3 Crystallographic data collection and refinement statistics**

| **Parameter** | **PahB–NAD⁺** | **PahB–NAD⁺–fluoranthene-7,8-dihydrodiol** |
| --- | --- | --- |
| **Data collection** |  |  |
| Space group | I222 | I222 |
| Unit cell (Å) | 74.54, 79.35, 94.94 | 74.84, 79.31, 94.43 |
| Angles (°) | 90, 90, 90 | 90, 90, 90 |
| Resolution range (Å) | 32.86–1.60 (1.66–1.60) | 39.67–1.35 (1.39–1.35) |
| Wavelength (Å) | 0.97853 | 0.97853 |
| Unique reflections | 37,495 (1,837) | 61,070 (2,863) |
| Completeness (%) | 100.0 (100.0) | 98.9 (94.7) |
| Redundancy | 13.0 (12.4) | 12.8 (9.5) |
| Mean I/σ(I) | 19.4 (3.4) | 23.5 (4.9) |
| Rmerge | 0.069 (0.809) | 0.070 (0.548) |
| Rpim | 0.020 (0.237) | 0.020 (0.182) |
| CC1/2 | 0.999 (0.883) | 0.999 (0.900) |
| **Refinement** |  |  |
| Reflections (work/free) | 35,592 / 1,900 | 54,961 / 2,972 |
| Rwork / Rfree | 0.1494 / 0.1680 | 0.1452 / 0.1595 |
| **No. of non-H atoms** |  |  |
| Protein | 1,993 | 1,946 |
| Ligands | 52 | 60 |
| Water | 271 | 312 |
| **B-factors (Å²)** |  |  |
| Protein | 28.01 | 16.34 |
| Ligands | 25.75 | 16.32 |
| Water | 38.44 | 30.22 |
| **R.m.s. deviations** |  |  |
| Bond lengths (Å) | 0.008 | 0.008 |
| Bond angles (°) | 1.095 | 1.145 |
| **Ramachandran plot (%)** |  |  |
| Favored | 97.69 | 97.69 |
| Allowed | 2.31 | 2.31 |
| Outliers | 0 | 0 |
